# Novel Nitrogen-Based Chalcone Analogs Provoke Substantial Apoptosis in HER2-Positive Human Breast Cancer Cells via JNK and ERK1/ERK2 Signaling Pathways

**DOI:** 10.3390/ijms22179621

**Published:** 2021-09-06

**Authors:** Balsam Rizeq, Ishita Gupta, Hadeel Kheraldine, Dana Elkhalifa, Halema F. Al-Farsi, Ala-Eddin Al Moustafa, Ashraf Khalil

**Affiliations:** 1 College of Medicine, QU Health, Qatar University, Doha P.O. Box 2713, Qatar; balsamr@gmail.com (B.R.); ishugupta28@gmail.com (I.G.); hk1805332@qu.edu.qa (H.K.); halfarsi@qu.edu.qa (H.F.A.-F.); 2 Biomedical Research Centre, Qatar University, Doha P.O. Box 2713, Qatar; 3 Biomedical and Pharmaceutical Research Unit, QU Health, Qatar University, Doha P.O. Box 2713, Qatar; 4 College of Pharmacy, Qatar University, Doha P.O. Box 2713, Qatar; 5 Department of Pharmacy, Aspetar Orthopedic and Sports Medicine Hospital, Doha P.O. Box 2713, Qatar; danaelkhalifa@gmail.com

**Keywords:** chalcones, HER2-positive, breast cancer, chemoprevention, apoptosis

## Abstract

Natural chalcones possess antitumor properties and play a role as inducers of apoptosis, antioxidants and cytotoxic compounds. We recently reported that novel nitrogen chalcone-based compounds, which were generated in our lab, have specific effects on triple-negative breast cancer cells. However, the outcome of these two new compounds on human epidermal growth factor receptor 2 (HER2)-positive breast cancer remains nascent. Thus, we herein investigated the effects of these compounds (DK-13 and DK-14) on two HER2-positive breast cancer cell lines, SKBR3 and ZR75. Our data revealed that these compounds inhibit cell proliferation, deregulate cell-cycle progression and significantly induce cell apoptosis in both cell lines. Furthermore, the two chalcone compounds cause a significant reduction in the cell invasion ability of SKBR3 and ZR75 cancer cells. In parallel, we found that DK-13 and DK-14 inhibit colony formation of both cell lines in comparison to their matched controls. On the other hand, we noticed that these two compounds can inhibit angiogenesis in the chorioallantoic membrane model. The molecular pathway analysis of chalcone compounds exposed cells revealed that these compounds inhibit the expression of both JNK1/2/3 and ERK1/2, the major plausible molecular pathways behind these events. Our findings implicate that DK-13 and DK-14 possess effective chemotherapeutic outcomes against HER2-positive breast cancer via the ERK1/2 and JNK1/2/3 signaling pathways.

## 1. Introduction

Breast cancer is one of the most significant public health impediments among females in the modern world and comprises about 25% of all cancer cases [1]. Based on gene expression patterns and hierarchical clustering, breast cancer is categorized into five molecular subtypes: Luminal (A and B), human epidermal growth factor receptor 2 (HER2), triple-negative and normal breast-like [2]. The luminal subtypes are hormone receptor (estrogen and progesterone)-positive and HER2 receptor-negative or -positive; while the HER2 subtype overexpresses the HER2 receptor and lacks hormone receptors [2,3]. On the other hand, the triple-negative breast cancer subtype lacks receptors for estrogen, progesterone or HER2 [2,3]. In addition, the normal breast-like subtype resembles the Luminal A subtype (hormone receptor-positive and HER2-negative) [2,3].

Of the four subtypes, approximately 20% of the total breast cancer cases are HER2-positive, where the tumors overexpress the human epidermal growth factor receptor type 2 (HER2) [4,5] and confer an aggressive tumor phenotype with a poor prognosis [6]. Chemotherapeutic agents can be classified according to their mechanism of action into drugs that prevent the growth of cancer cells, and others that cause DNA damage that leads to apoptosis [7]. For HER2-positive breast cancer, the recommended first-line treatment consists of trastuzumab in combination with cytotoxic agents, i.e., a taxane such as paclitaxel or docetaxel, considering a better tumor response, progression-free survival and overall survival compared with the cytotoxic agent alone [8]. Conventional approaches are sometimes inconvenient and unsatisfactory due to drug resistance [9], adverse side effects and poor selectivity [10]. Therefore, treatment of breast cancer requires novel drug compounds with low resistance as well as a high-potential treatment ability, minimum side effects, and high selectivity to their target mutated cells, which are considered as the initiating point of cancer. For these reasons, the synthetic modification of novel drugs may improve their potency as anti-cancer agents; these can be either natural or synthetic, and are categorized as chemo-preventive agents [11,12]. Chemo-preventive agents include chalcones and their derivatives.

Chalcone is an aromatic ketone that forms the central core for a variety of critical biological compounds [13]. Chalcones are essential intermediates in the synthetic pathways of flavonoids; they possess a wide variety of therapeutic activities and can also serve as versatile starting materials for various complex molecules in medicinal chemistry [14]. Recent studies have shown that chalcones exhibit a wide variety of biological activities against cancer [15,16], bacteria [17], fungi [18], and viruses [19]. They also exhibit antioxidant [20], anti-depressant [21], and anti-inflammatory [22] activities, and can potentially reduce resistance to various chemotherapeutic agents [23]. More specifically, chalcones inhibit the development and progression of cancer, suggesting that chalcones and their derivatives can act as promising candidates against cancer, including breast cancer [24,25]. Hence, investigators are generating a wide range of chalcones to explore their efficacy in various therapeutic areas.

We recently reported that the chalcone-based compounds DK-13 and DK-14, developed by our group, impair the growth of triple-negative breast cancer [24]; thus, we herein explore the outcome of these two new compounds on two HER2-positive breast cancer cell lines in addition to angiogenesis of the chorioallantoic membrane (CAM) model and their underlying mode of action.

## 2. Results

**Figure 1:**
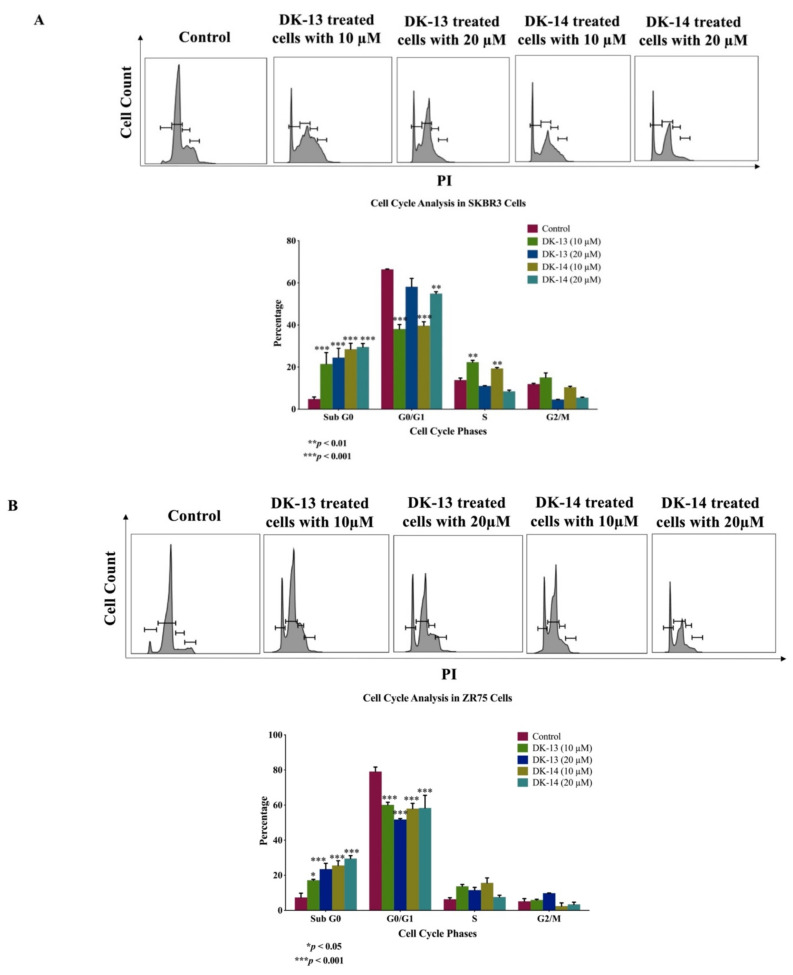
(**A,B**). Cell cycle flow cytometry analysis of HER2-positive breast cancer cell lines, (**A**) SKBR3 and (**B**) ZR75. Cells were incubated with 10 and 20 µM of chalcone compounds, DK-13 or DK-14, for 48 h followed by staining of cells with propidium iodide (PI). Representative DNA content histogram showing the phases of sub G0, G0\G1, S, and G2/M on tested cell lines upon treatment with chalcone compounds, DK-13 and -14 compared to controls. The cell cycle histogram results reveal that chalcone compounds, DK-13 and -14 at lower concentrations (10 µM) can disrupt cell mitosis by arresting cells in the S phase and at higher concentrations (20 µM) induce apoptosis. Quantification is represented as the mean ± SEM (*n* = 3).

In this study, the DK-13 and 14 chalcone compounds, which were recently developed by our group, were evaluated for their antitumor activities on the HER2-positive breast cancer cell lines SKBR3 and ZR75 after 48 h of treatment. Cells were treated with DK-13 and -14 compounds at different concentrations ranging from 5 to 60 μM; we found that DK-13 and -14 inhibit cancer cells proliferate in a dose-dependent manner; notably, treatment with DK-13 (20 µM) reduced the viability of both cell lines by ~50%, whereas treatment with DK-14 (20 µM) reduced the viability of cells by 60% and 70% in SKBR3 and ZR75 cells, respectively, in comparison with their matched control. Based on these data, concentrations of 10 and 20 μM were selected for further investigation in both cancer cell lines. We found that 10 and 20 μM concentrations of DK-13 and -14 slightly affect the viability of human normal mammary epithelial cells immortalized by E6/E7 of HPV type 16 (Data not shown). Meanwhile, to validate the antiproliferative effect of our two compounds on SKBR3 and ZR75 cells, we analyzed the cell-cycle phase distributions of chalcone compounds-treated cells using flow cytometric analysis. As shown in Figure 1, the control group displays a typical cell cycle pattern in G0/G1, S, and G2/M. On the other hand, treatment of both SKBR3 and ZR75 with 10 µM of DK-13 or -14 induced a significant cell cycle arrest at the S phase (*p* < 0.001) (Figure 1). However, in comparison to the control, when cells were treated with 20 µM of DK-13 or 14, a significant increase in the sub-G0/G1 phase was noted, which is a marker of apoptosis (Figure 1). The cell cycle histogram results reveal that both chalcone compounds (DK-13 and -14) have the ability to interrupt cell mitosis by arresting SKBR3 and ZR75 cells in the S phase at 10 µM concentration and induce apoptosis at higher concentrations (20 µM), leading to impaired cancer cell proliferation (Figure 1).

**Figure 2:**
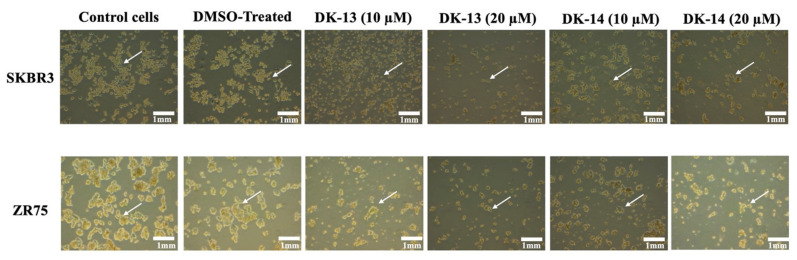
Effect of cell morphology on chalcone compounds DK-13 and 14 in the HER2-positive breast cancer cell lines, SKBR3 and ZR75. We observe that treatment for 48 h with 10 and 20 μM of compounds DK-13 and 14 induces cell death and the formation of a monolayer of cells in both cell lines (white arrows indicate loss of cell-cell adhesion), in comparison with untreated (control) and DMSO-treated (negative control) cells, which show no cytotoxic effect, display a round phenotype and form multilayers; white arrows indicate epithelial morphology with clear cell-cell adhesion.

Next, we analyzed the cell morphological characteristics of SKBR3 and ZR75, using phase-contrast microscopy, under the effect of 10 and 20 μM of chalcone compounds (DK-13 and -14, respectively). In the absence of treatment (control), SKBR3 and ZR75 cells displayed a round morphology and disorganized multilayered cells (Figure 2). After treatment for 48 h with chalcone compounds, the presence of DK-13 or -14 led to morphological changes including deformation, contact inhibition, condensed nuclei, apoptotic bodies, cell shrinkage, and reduced numbers of viable cells (Figure 2). Our results indicate that chalcone compounds (DK-13 and -14) can affect cell morphology by the induction of cell death.

**Figure 3:**
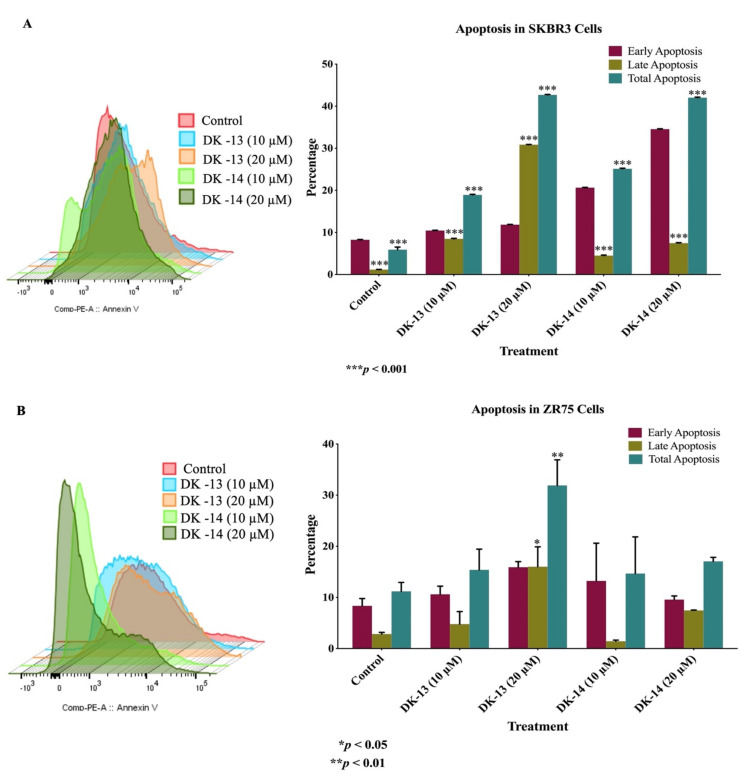
(**A**,**B**). Impact of chalcone compounds (DK-13 and -14) on cell apoptosis at quantities of 10 and 20 µM in (**A**) SKBR3 and (**B**) ZR75 cell lines.

To further evaluate whether the chalcone compounds DK-13 and -14 could induce cell death, Annexin-V-FITC/7-AAD staining by flow cytometry was performed to evaluate the apoptotic impact of the tested chalcone compounds, compared to untreated cells (control). After treatment of SKBR3 and ZR75 cells with chalcone compounds (DK-13 and -14) at the concentrations of 10 and 20 µM for 48 h, DK-13 was more effective than DK-14 in inhibiting the proliferation of HER2-positive breast cancer cell lines, especially in late the apoptotic phase (Figure 3) (Supplementary Figure S1). Compared to the control group, our results in Figure 3 show that the chalcone compounds DK-13 and -14 significantly induce early and late apoptosis.

**Figure 4:**
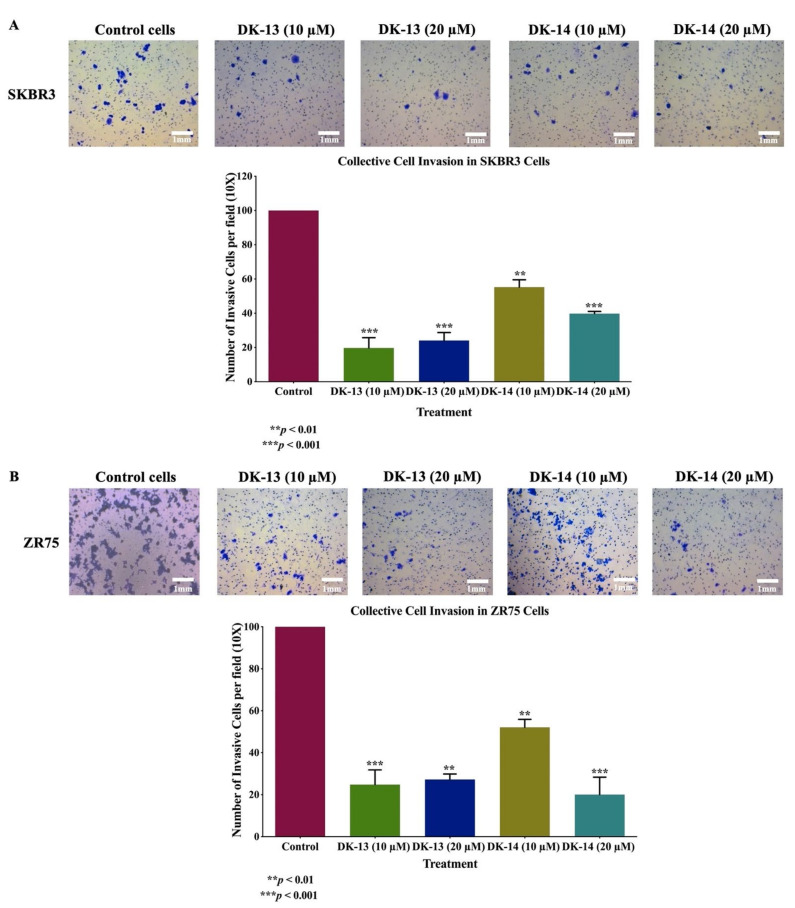
(**A**,**B**)**.** Effects of compounds DK-13 and -14 on cell invasion of human HER2-positive breast cancer cells. Chalcone compounds (DK-13 and -14) inhibit cell invasion ability of (**A**) SKBR3 and (**B**) ZR75 cell lines using Boyden chambers. Cancer cells treated for 48 h with 10 and 20 µM chalcone compounds show a significant inhibition of cell invasion in both cell lines, when compared with their matched control (** *p* < 0.01, *** *p* < 0.001). Data are quantified by normalizing the number of invasive cells by their total number.

Next, to examine the invasion effects of DK compounds -13 and -14 on SKBR3 and ZR75 cell lines, we performed the Matrigel invasion assay. Upon treatment with 10 and 20 μM of compounds DK-13 and -14, our data revealed that treatment with DK-13 at 10 and 20 μM concentrations reduced the invasion ability of both SKBR3 and ZR75 cells in a dose-dependent manner, with a significant reduction of 80.3% and 76%, respectively, in comparison to the control (Figure 4A). On the other hand, treatment with DK-14 at 10 and 20 μM inhibited invasion by 44.7% and 60.2%, respectively, in both cell lines, as compared to the control (Figure 4B). Collectively, our results indicated that treatment of HER2-positive breast cancer cell lines (SKBR3 and ZR75) with compounds DK-13 and -14 inhibits cell invasion, thereby attenuating breast cancer progression through the impairment of cell invasion.

**Figure 5:**
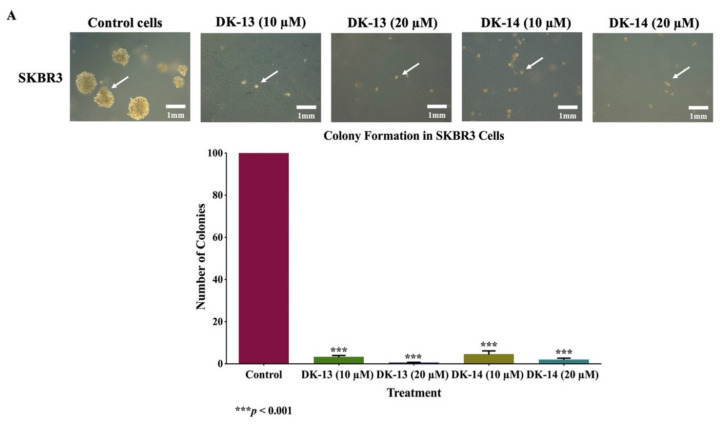

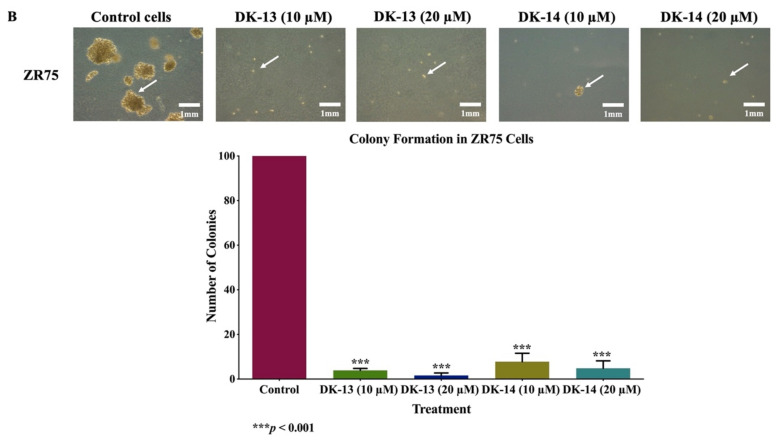
(**A**,**B**). Effect of chalcone compounds (DK-13 and -14) on colony formation, in soft agar, in human HER2-positive cancer cell lines, (**A**) SKBR3 and (**B**) ZR75. The compounds inhibit colony formation of SKBR3 and ZR75, in comparison with their matched control cells; as marked by the white arrows (×10 magnification). The colonies were counted manually and expressed as percentage of treatment relative to the control (mean ± SEM).

Furthermore, we analyzed the colony formation of SKBR3 and ZR75 cells, in soft agar, under the effect of chalcone compounds (DK-13 and -14) at 10 and 20 µM, for two weeks; we observed a significant decrease in the number of colonies for both cell lines treated with chalcone compounds, in comparison with their matched control, as shown in Figure 5. DK-13 treatment of SKBR3 significantly reduced colony formation by 96.6% (*p* < 0.001) and 99.4% (*p* < 0.001) when exposed to 10 and 20 µM concentrations, respectively, relative to the control (Figure 5A). On the other hand, DK-14 treatment inhibited colony formation in SKBR3 cells by 95.4% (*p* < 0.001) and 98.0% (*p* < 0.001) when exposed to 10 and 20 µM, respectively, relative to untreated control (Figure 5A). In parallel, ZR75 cells also showed a similar pattern after two weeks of treatment; in comparison to the control, the number of colonies decreased by 96.1% (*p* < 0.001) and 98.4% (*p* < 0.001) at 10 and 20 µM of DK-13, respectively (Figure 5B), while DK-14 treatment at 10 and 20 µM concentrations decreased the number of colonies by 92.2% (*p* < 0.001) and 95.2% (*p* < 0.001), respectively (Figure 5B). Our results indicate that the loss of colony-forming ability in both of the HER2-positive breast cancer cells upon treatment with the chalcone compounds DK-13 or -14, compared to the corresponding controls, indicates the tumor suppressor ability of these compounds in in vivo.

**Figure 6:**
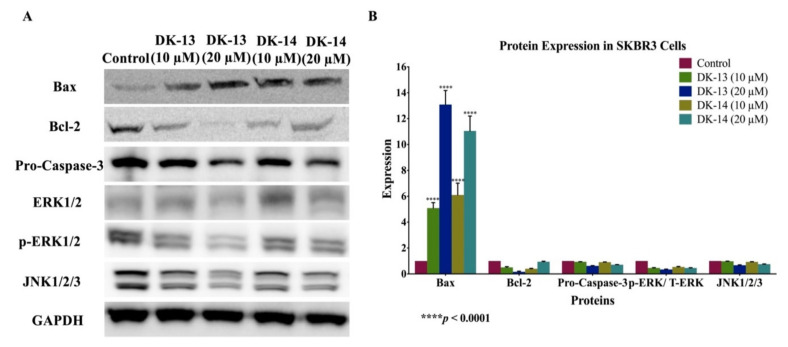
(**A**,**B**). Protein expression and molecular mechanisms of chalcone compounds’ (DK-13 and -14) inhibitory actions in SKBR3 cell line. Chalcone compounds induce the expression of pro-apoptotic markers Bax and Caspase-3 in comparison with their control, while anti-apoptotic marker Bcl-2 is inhibited. Furthermore, these compounds both inhibit the expression of JNK1/2/3 and enhance ERK1/2. GAPDH was used as a control in this assay. Cells were treated with 10 and 20 μM of DK-13 and -14 compounds for 48 h, as explained in the Materials and Methods section. (**A**) Blot image and (**B**) quantification of bands.

**Figure 7:**
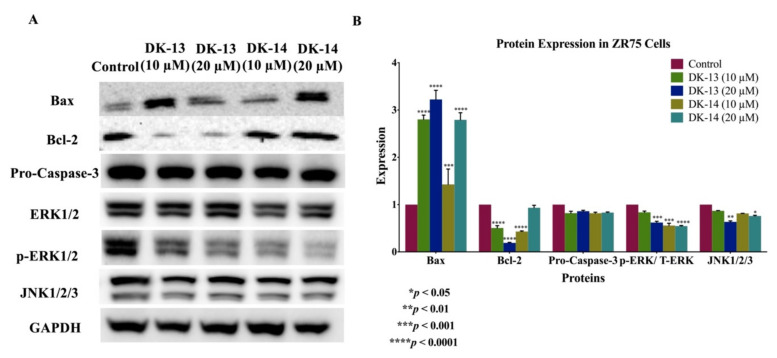
(**A**,**B**). Protein expression and molecular mechanisms of chalcone compounds (DK-13 and DK-14) inhibitory actions in ZR75 cell line. These compounds induce the expression of pro-apoptotic markers Bax and Caspase-3 in comparison with their control, while anti-apoptotic marker Bcl-2 is inhibited. Furthermore, DK-13 and -14 inhibit the expression of both, JNK1/2/3 and ERK1/2. GAPDH was used as a control in this assay. Cells were treated with 10 and 20 μM of DK-13 and -14 compounds for 48 h, as explained in the Materials and Methods section. (**A**) Blot image and (**B**) quantification of bands.

Based on the data above, since the chalcone compounds (DK-13 and -14) exhibited cell apoptosis abilities, we examined the expression patterns of key markers of apoptosis in HER2-positive breast cancer cells (SKBR3 and ZR75) using western blot analysis. The data show that DK-treated cells enhance the expression of the pro-apoptotic marker Bax, while the expression of the anti-apoptotic marker Bcl-2 was lost in both cell lines (Figure 6 and Figure 7). Moreover, in comparison to the control, pro-caspase-3 expression was downregulated on exposure to the DK-chalcone compounds (Figure 6 and Figure 7). Our data clearly suggest that high concentrations of the DK-compounds induce apoptosis in HER2-positive cancer cells, which is associated with the deregulation of Bcl-2/Bax/caspase-3. Regarding the underlying molecular pathways of our new compounds on cell apoptosis, as well as other events provoked in SKBR3 and ZR75 cell lines in addition to the inhibition of angiogenesis, we postulate that c-Jun N-terminal kinase (JNK) and ERK1/2 could play vital roles in the regulation of these events; thus, we examined the expression patterns of JNK1/2/3 as well as ERK1/2. Our data show that treatment with DK-13 and 14 inhibits the expression of JNK1/2/3 as well as the expression of total and phosphorylated ERK1/2 in both HER2-positive breast cancer cell lines in comparison with their controls (Figure 6 and Figure 7).

**Figure 8:**
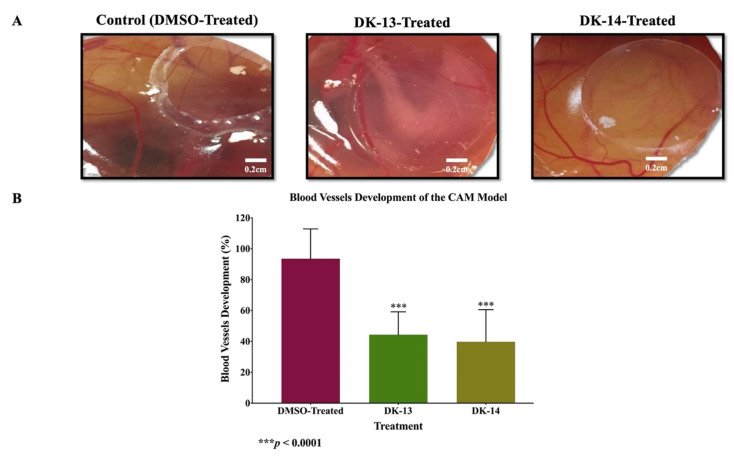
(**A**,**B**). The effect of DK-13 and -14 on angiogenesis of the CAM model 48 h after treatment. This analysis was performed using CAM treated with DMSO (control) and those exposed to chalcone compounds (DK-13 and DK-14). (**A**) The images on the top show the unexposed areas and those on the bottom show areas that were exposed to the treatment (under the coverslip) of the same embryo. (**B**) Two areas within each individual embryo in both groups were compared to examine total blood vessels’ development of controls vs. treated embryos (*** *p* < 0.0001).

Finally, based on the fact that angiogenesis plays an important role on cancer progression, we investigated the effect of DK-13 and -14 on blood vessel development in vivo using the CAM of the chicken embryo, as described in the Materials and Methods section. Here, it is important to highlight that 10 and 20 µM concentrations were selected based on our recent published work on this topic [24]. The compounds DK-13 and 14 significantly inhibit blood vessels’ development in the CAM model as compared to the control (55.6 ± 6.04% and 60.20 ± 8.47% reduction, respectively, with *p*-value of < 0.0001) (Figure 8). The data indicate that compound DK-14 has a significant advantage over DK-13 in the event.

## 3. Discussion

In this study, we explored the outcome of nitrogen-based chalcone analogs (compounds DK-13 and -14) in two HER2-positive human breast cancer cell lines, SKBR3 and ZR75 on certain parameters related to cell proliferation, cell cycle, apoptosis, cell invasion and colony formation in addition to angiogenesis. We also investigated the underlying mechanism of action of these drugs and their molecular pathways. The DK-13 and -14 compounds were recently generated and evaluated for their effect on triple-negative breast cancer (TNBC) cell lines by our group [24]. While, we herein report that these compounds inhibit cell proliferation, deregulate the cell cycle, reduce cell invasion and colony formation of both HER2-positive breast cancer cell lines. More significantly, the two compounds induce cell apoptosis in these cell lines. However, it is important to highlight that compound DK-13 has a significant advantage over DK-14 in certain parameters while having a minimal effect on the growth of normal immortalized mammary epithelial cells. 

In our recent work regarding the outcome of DK-13 and -14 in TNBC cell lines, we reported that DK-14 has a significant effect on cell differentiation and invasion of TNBC cells in comparison to DK-13 [24]. We noted that DK-14, but not DK-13, induce mesenchymal to epithelial transition (MET) of two TNBC cell lines in comparison with their control [24]. However, we herein reveal that DK-13 has a more pronounced effect on HER2-positive cell lines in comparison with DK-14, which is not accompanied by MET (differentiation) rather its mechanism of action seems to be apoptotic induction.

In this regard, DK-chalcone based compounds blocked the proliferation of HER2 positive breast cancer cells ZR75 and SKBR3 by inducing apoptosis, combined with sub G0 phase arrest and S phase blockade. The first study by Peyrot et al., (1989) reported an antimitotic role of chalcones; the group demonstrated that chalcones result in G2/M phase arrest during cell cycle progression [26]. In addition, similar to our data, other studies have reported chalcone targets that induce apoptosis along with hindering cell cycle progression, these mechanisms were mediated by loss of cyclin expression [27,28] or induced expression of p21, an inhibitor of CDKs [28,29]. Moreover, chalcones were found to downregulate cyclin D1 and enhance p53 expression, inducing G2/M phase arrest [30]. Similar to our data, the chalcone compound L2H17 showed selective cytotoxic effect on mouse colon cancer cells (CT26.WT) by arresting G0/G1 phase and apoptosis [31]. A trimethoxyphenyl-derived chalcone-benzimidazolium salt, compound 7f, provoked G1 phase arrest and apoptosis in the myeloid liver carcinoma cells, SMMC-7721 [32]. In addition, 3-Phenylquinolinylchalcone derivatives triggered antiproliferative activities and G2/M arrest in the non-small cell lung cancer (H1299) and HER2-positive breast cancer cells (SKBR-3), which is in concordance with our data [33]. β-Carboline based chalcones showed cytotoxic activity in the breast cancer cell line, MCF-7 [34]. Moreover, the 2′-Hydroxy-2,4,6-trimethoxy-5′,6′-naphthochalcone, 8 (chalcone 8) induced cell cycle arrest at G2/M phase, followed by an increase in apoptotic cell death in colon cancer cells, SW620 [35]. In cervical cancer cells (HeLa and C33A), the anthraquinone based chalcone compounds blocked cell proliferation by arresting G2/M phase and triggering apoptosis [36]. On the other hand, another study by Park et al., showed that cardamonin, a chalcone, inhibited cell proliferation in colon cancer by downregulating β-catenin, cyclin D1 and c-myc levels [37]. In lung cancer, isoxazolyl chalcones increased the percentage of cell in sub-G0 phase and decreased the percentage of cell in G0/G1 phase, indicating the presence of apoptosis [38]. 

In the present study, we demonstrate that DK-13 and -14 can hinder cell migration of both SKBR3 and ZR75 cell lines. Moreover, this data is in accordance with our recently published work regarding the effect of chalcone analogs in TNBC cells [24]. Similar to our data, the trans-chalcone, xanthohumol inhibited invasion of breast cancer cells, MCF-7/6 and T47-D by restoring the function of E-cadherin/catenin complex [39]. In addition, L2H17, a chalcone compound inhibited both, migration and invasion of the mouse colon cancer cells, CT26.WT, in addition to the expression of E-cadherin [31]. A recent study demonstrated that a chalcone inhibited growth, migration and invasion in esophageal cancer cells (KYSE-4) by inhibiting the Wnt/β-catenin signaling [40]. On the other hand, we found that DK-13 and -14 inhibit the colony formation of HER2-positive breast cancer cell lines, which is an indicator of tumor growth in in-vivo. While, in our recent work, we noted that DK-13 and -14 also block colony formation of TNBC cell lines [24]. Thus, and based on our above data, we believe that these DK-chalcone compounds can be plausible candidate alternatives in the management of HER2-positive breast cancer.

In parallel, and based on the fact that angiogenesis plays an important role in cancer progression, we explored the effect of DK-13 and -14 on blood vessel development using the CAM of the chicken embryo model; we found that both compounds inhibit angiogenesis of the CAM. Several studies have reported the effect of chalcones on angiogenesis and neovascularization. Studies showed treatment with chalcones reduced expression of several cell adhesion molecules including VCAM and ICAM, thus suggesting that chalcones inhibit angiogenesis in addition to the formation of tube-like structures, both at intracellular and extracellular levels [41,42,43,44,45]. Also, chalcone exposure led to loss of VEGF-R2 signaling along with VEGF-stimulated tumor growth in two xenograft models due to reduced vessel density [46]. Similarly, series of quinolyl-thienyl chalcones were identified as candidate VEGFR-2 kinase inhibitors [47]. Moreover, synthetic chalcone analogue compounds were found to reduce MMP-9 and VEGF secretion, thus, inhibiting VEGF-induced migration of HUVECs [43]. Both in-vitro and in-vivo studies have shown xanthohumol to possess antiangiogenic property [41,48,49]. Imine derivatives of hybrid chalcone analogues (13a, 13b, 13c, and 13d) containing anthraquinone scaffold exhibited significant anti-tubulogenic and anti-angiogenic effect against HeLa, LS174, and A549 cancer cells by inhibiting matrix metalloproteinase-2 (MMP-2) secretion [50]. Combretastatin analogues, like colchicine are commonly reported to target angiogenesis and tumor vasculature; the combretastatin CA-4P is under advanced stages of cancer clinical trials [51,52].

Based on the above data, we studied the outcome of DK-13 and -14 on key apoptotic genes’ regulation, we analyzed the expression patterns of mitochondrial apoptosis (Bcl-2 and Bax), as well as caspase-3 [53]; we found that DK-13 and -14 enhance the expression of Bax, while pro-caspase-3 and Bcl-2 is inhibited. Studies have reported chalcones to possess apoptotic properties; they generally induce apoptosis via the mitochondrial pathway. Similar to our data, various studies demonstrated upregulation of the pro-apoptotic proteins Bax [54,55,56] and Bak [54,55] with downregulation of Bcl-2 [54,55,57] and Bcl-x [54,55,57]. Concordantly, in leukemia and cervical cancer cells, exposure to naphthyl chalcones [58], 2′,4′,5′-trimethoxychalcones [59] as well as coumarin-chalcone hybrids [36] induced apoptosis via the caspase-dependent pathway; the chalcones reduced Bcl-2 expression, while Bax and caspase-3 expression was upregulated, thus triggering the intrinsic pathway and inducing apoptosis. 

Finally, and regarding the molecular pathways underpinning the role of chalcone-compounds on HER2-positive breast cancer cells, we analyzed expression of extracellular signal regulated kinase 1/2 (ERK1/2) and c-Jun N-terminal kinase (JNK). MAPKs are widely known to regulate cellular processes including cell survival, proliferation and apoptosis [60,61]. ERK1/2 is associated with cell survival and proliferation [62]. Our data shows that after 48h of DK-13 and -14 treatment the expression of ERK1/2 and phosphor-ERK1/2 is inhibited. Previous studies have shown chalcones to reduce levels of phosphorylated Erk1/2 [63,64]; our data correlate with the induction of apoptosis. Moreover, apoptosis and tumor cell survival and invasion correlate with JNK activation [65]. In this study, we showed that exposure to DK-13 and -14 inhibit JNK expression; the data is concordant with a study where exposure to chalcone L1 increased levels of phosphorylated-JNK [64]. Concordant to our data, exposure to chalcone analog (chalcone-24) enhanced activated caspase-3 expression, and inhibited activation of ERK1/2 and JNK in lung cancer cells (A549) [66]. Moreover, a recent study by Sian et al., demonstrated that a chalcone derivative suppressed cytokine secretion by inhibition of ERK and JNK pathways [67]. This confirms the role of the chalcone analogues, DK-13 and -14 in promoting cell cycle arrest, inhibiting proliferation, and inducing apoptosis by modulating the components of MAPK signaling pathways.

Overall, these observations endorse that DK chalcone based compounds, especially DK-13, might be considered as a potential treatment modality for HER-2 positive breast cancer. In addition, it is important to determine whether DK chalcone based compounds inhibit autophagy and/or modify cellular bioenergetics. Thus, further studies will involve determining autophagy inhibition as well as in-vivo studies to determine their impact on tumor growth using orthoptic xerograph and/or neu-transgenic mouse models. Interestingly, these findings suggest that DK compounds have a selective therapeutic profile against TNBC and HER2-positive breast cancer, which requires further investigation to confirm and determine their specific mechanism of action. Therefore, we believe it is important to explore the outcome of these compounds on other types of human carcinomas.

## 4. Materials and Methods

### 4.1. Compounds Preparation

The compounds DK-13 and DK-14 were prepared as recently described by our group [24].

### 4.2. Cell Culture

HER2-positive breast cancer cell lines (SKBR3 and ZR75) were obtained from the American Type Tissue Culture (ATCC, Manassas, VA, USA). Cell lines were grown and cultivated in Gibco^®^ RPMI-1640 (Gibco, Life Technologies, Waltham, MA, USA) supplemented with 10% heat-inactivated fetal bovine serum (PAN-Biotech, Aidenbach, Germany), 1X antibiotics (PEN.STREP) (Invitrogen, Waltham, MA, USA) in a humidifying incubator of 37 °C with CO_2_ concentration of 5%. To assess chalcone compounds’ safety, human normal mammary epithelial (HNME) cells immortalized with the E6/E7 gene of HPV type 16 (HNME-E6/E7), which were previously established by our group [68], were used and maintained in Keratinocyte-SFM (KSFM) (1X) media with 100 µg/mL penicillin–streptomycin (Gibco, Life Technologies, Waltham, MA, USA).

### 4.3. Cell Viability Assay

Chalcone compounds, DK-13 and 14 were generated and evaluated as recently described by our group [24]. SKBR3, ZR75 and HNME-E6/E7 cells were seeded at a concentration of 3 × 10^5^ cells/well in 6-wells plates and allowed to attach overnight. The following day, cells were treated with DK-13 and DK-14 compounds at different concentrations ranging from 5 μM to 60 μM. At 24 and 48 h, cell morphology was evaluated and assessed under an inverted light microscope (Leica, Wetzlar, Germany).

### 4.4. Cell Cycle

SKBR3 and ZR75 cells (1 × 10^6^) were seeded into 100 mm plates and maintained in culture overnight. The following day, cells were starved overnight with serum-free media for a period of 12 h before treatment. Subsequently, cells were treated with chalcone compounds (DK-13 and DK-14) for 48 h and then treated cells were collected, washed with phosphate-buffer saline and fixed with 70% ice-cold ethanol. Following fixation, cells were collected, washed with ice-cold PBS, suspended in FXCycle propidium iodide (50 μg/mL) and 0.5 mL of RNase (50 μg/mL), then incubated at 37 °C, according to the manufacturer’s instructions (Invitrogen, Thermo Fisher Scientific Waltham, MA, USA). Flow cytometry (BD Accuri C6, BD Biosciences, Franklin Lakes, NJ, USA) was used to analyze the cells. The FlowJo V10 software was used to quantify the cells at different phases of the cell cycle.

### 4.5. Annexin V Apoptosis Assay

We analyzed cell apoptosis using the AnnexinV/fluorescein isothiocyanate (FITC)/7-amino-actinomycin D (7-AAD) Apoptosis Kit (559763BD/Biosciences, Franklin Lakes, NJ, USA) to assess the effect of the chalcone compounds (DK-13 and 14) on SKBR3 and ZR75 cells at 48 h after treatment, as previously described by our group [69]. In brief, cells were trypsinized, washed, and resuspended in 1X binding buffer. Each group of cells were incubated with Annexin V-FITC (5 µL) and/or 7-AAD (5 µL) following the manufacturer’s recommendation (Clontech, Palo Alto, CA, USA). On the other hand, the controls (unstained cells) were stained with PE Annexin V (no 7-AAD) as well as 7-AAD (no PE Annexin V). Samples were incubated in the dark for 15 min and analyzed by flow cytometry (BD Accuri C6, BD Biosciences, Franklin Lakes, NJ, USA). The FlowJo V10 software was used to quantify and analyze the results. The data were depicted as density plots of Annexin V-FITC and 7-AAD staining.

### 4.6. Cell Invasion Assay

Cell invasion assays were carried out in 24-well Transwell chambers (8 μm pore size, BD Biosciences, Oxford, UK) as recommended by the manufacturer. In brief, the bottom chamber was filled with RPMI-1640 medium and 5 × 10^4^ cells/well (treated and untreated) were seeded in the upper compartment and incubated at 37 °C to allow the cells to migrate for 48 h. Cells in the upper chamber were mechanically removed with a cotton swab and migrated cells were fixed with methanol and stained with 0.4% crystal violet. Cells were counted under an inverted microscope (Leica DMi1, Leica Microsystems, Wetzlar, Germany) in five predetermined fields and quantification was performed as previously described [68]. The percentage of inhibition of migrated cells was calculated with respect to untreated cells. Each experiment was carried out in triplicate.

### 4.7. Soft Agar Colony Formation Assay

Colony formation in soft agar was used to determine cells’ capacity to colonize in vitro. A total of 2 × 10^4^ cells of SKBR3 and ZR75 were placed in RPMI medium containing 0.2% agar with/without DK-13 and 14 (10 and 20 μM) (treated and control cells, respectively) and plated in a 6-well plate covered with a layer of 0.4% noble agar in RPMI complete growth media (1 mL solid agar layer/well). Colony formation was monitored every 2 days up to three weeks, and pictures of the colonies were taken from various locations in each well using the inverted light microscope (Leica, Wetzlar, Germany).

### 4.8. Western Blotting

We examined the expression levels of different genes using western blot analysis, as recently shown by our group [69]. Briefly, SKBR3 and ZR75 (1 × 10^6^ cells) were seeded and treated with the chalcone compounds DK-13 or DK-14 at 10 μM and 20 μM concentrations for 48 h. Cell lysates were harvested, and an equivalent protein concentration of 30 μg was separated on gradient polyacrylamide gels (PAGE) and blotted onto nitrocellulose membranes followed by probing with the following primary antibodies: anti-mouse Bax (Thermo Fisher Scientific, Mississauga, ON, Canada), anti-mouse Bcl-2, anti-rabbit Pro-caspase-3, anti-rabbit anti-ERK1/ERK2 antibody, anti-rabbit phosphorylated ERK1/ERK2 and anti-rabbit JNK1/JNK2/JNK3 (Abcam, Cambridge, MA, USA). To ensure equal protein loading in all samples, re-probing of membranes was undertaken with the housekeeping protein anti-rabbit GAPDH (Abcam, Cambridge, MA, USA).

Different protein bands were developed by chemiluminescence ECL-Western blotting (Pierce Biotechnology, Waltham, MA, USA) as described by the manufacturer. For relative quantification of protein expressions, ImageJ software was used to analyze the acquired western blotting images. Bands’ intensities normalized to β-actin was used to determine the relative protein expression in each cell line.

### 4.9. Angiogenesis Assay

The chorioallantoic membrane (CAM) of the chicken embryos was treated on day five of incubation to evaluate the outcomes of 5 and 10 mM of DK-13 and 14, individually, on the vascular development of the CAM. The prepared compound was placed on a circular glass cover slip for 24 and 48 h, respectively, as previously performed by our group [70,71]. DMSO-treated embryos were used as controls. After 24 and 48 h of treatment, the vascular development of the CAM was examined daily over a period of three days under a stereomicroscope. Images were captured and the number of branching points and length of blood vessels were quantified using the AngioTool Software 0.6a [72]. Briefly, images were extracted to this program. All extracted images have the same size and magnification with unified AngioTool Software 0.6a inputs.

### 4.10. Statistical Analysis

All the statistical data analysis was evaluated using the GraphPad Prism software (version 8.4.3). To compare the difference between treated and untreated cells, the statistical *t*-test was used. Differences between chalcone compounds and controls were assessed using One-way (ANOVA) followed by Tukey’s posthoc test. For cell cycle and apoptosis experiments, two-way ANOVA and the Benferonni posthoc test were used. Results were presented from three independent experiments as mean ± SEM and data with *p* < 0.05 were considered statistically significant.

## 5. Conclusions

In this study, we report the effect of chalcone-based compounds in HER2-positive breast cancer and its underlying mechanism. In an effort to explore innovative selective treatments against HER2-positive breast cancer, and in continuation of our previous work on chalcone-based nitrogen analogs against TNBC, we herein point out that compounds DK-13 and -14 are promising therapeutic agents against certain types of breast cancer, since they induce JNK activation and significantly hinder the proliferation of breast cancer cells. Moreover, our data also signify that these compounds stimulate apoptosis, particularly in HER2-positive breast cancer cells, which is associated with the Bcl-2/Bax/caspase-3 signaling pathway. Subsequently, compound DK-13 was recognized as the most promising candidate for further investigation as it has the greatest anti-cancer potential against HER2-positive breast cancer. Thus, further investigations are necessary to evaluate the complete effect of these compounds on different subtypes of breast cancer in addition to exploring their role in other types of human carcinomas. This could pave the way for advanced therapeutic approaches in human breast cancer management, especially in HER2-positive cases in addition to other types of cancers.

## Data Availability

The data presented in this study is contained within the article or the Supplementary Material.

## Supplementary Materials

The following are available online at https://www.mdpi.com/article/10.3390/ijms22179621/s1.
