# Supplementary material for: Novel Nitrogen-Based Chalcone Analogs Provoke Substantial Apoptosis in HER2-Positive Human Breast Cancer Cells via JNK and ERK1/ERK2 Signaling Pathways
Source: Int J Mol Sci. 2021 Sep 6;22(17):9621. doi: 10.3390/ijms22179621 (PMC8431802; doi:10.3390/ijms22179621)
Supplement: Supplementary file 1 [file ijms-22-09621-s001.zip › ijms-1292807-Supplementary.pdf]

A

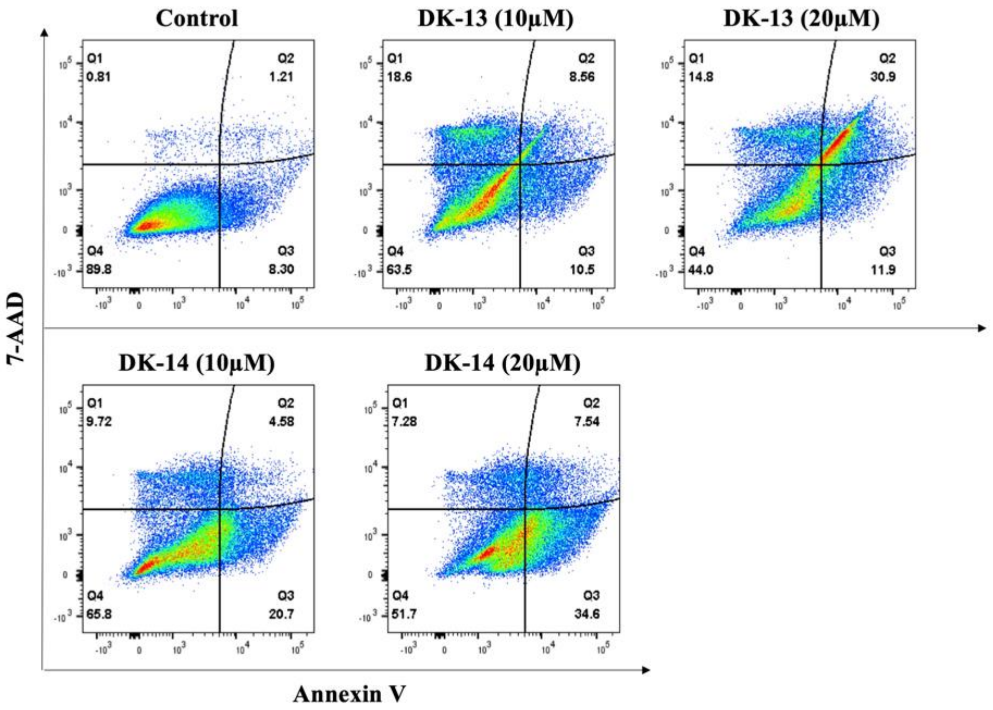

B

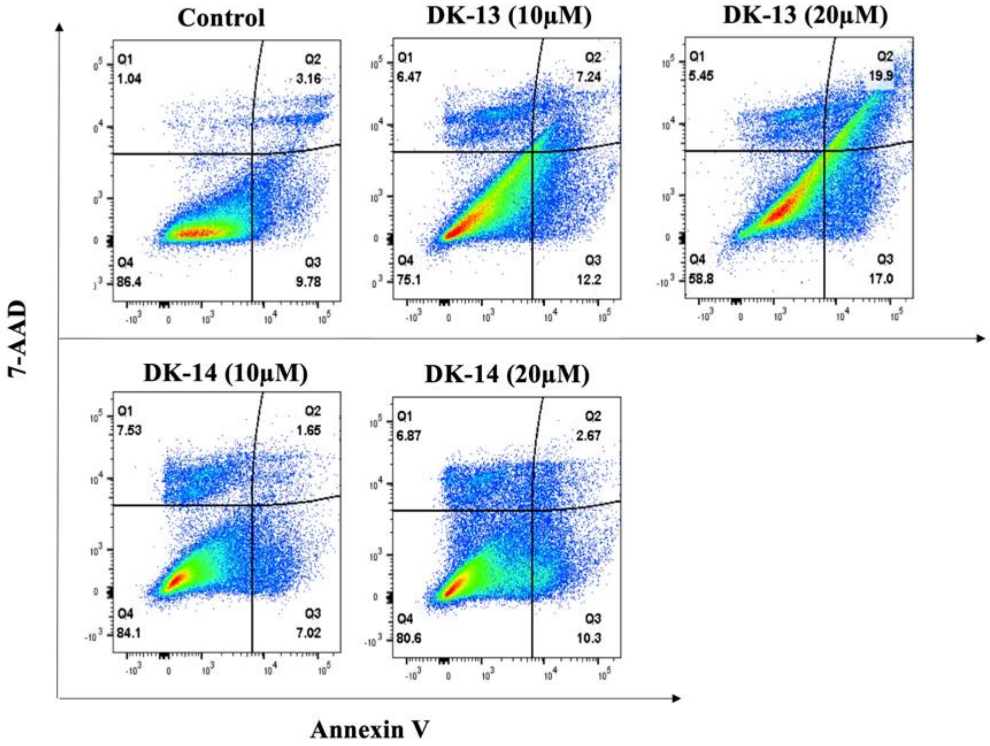

Figure S1. Induction of apoptosis by DK-13 and DK-14 chalcone compounds in (**A**) SKBR3 and (**B**) ZR75 cells as determined by Annexin V apoptosis assay.
